# Immunological Aspect of Radiation-Induced Pneumonitis, Current Treatment Strategies, and Future Prospects

**DOI:** 10.3389/fimmu.2017.00506

**Published:** 2017-05-02

**Authors:** Anup Kainthola, Teena Haritwal, Mrinialini Tiwari, Noopur Gupta, Suhel Parvez, Manisha Tiwari, Hrideysh Prakash, Paban K. Agrawala

**Affiliations:** ^1^Department of Radiation Genetics and Epigenetics, Institute of Nuclear Medicine and Allied Sciences, Delhi, India; ^2^Department of Toxicology, School of Chemical and Life Sciences, Jamia Hamdard University, New Delhi, India; ^3^Ambedkar Center for Biomedical Research, University of Delhi, Delhi, India; ^4^School of Life Sciences, Science complex, University of Hyderabad, Hyderabad, India

**Keywords:** radiation pneumonitis, fibrosis, lungs, inflammation, HDAC inhibitor

## Abstract

Delivery of high doses of radiation to thoracic region, particularly with non-small cell lung cancer patients, becomes difficult due to subsequent complications arising in the lungs of the patient. Radiation-induced pneumonitis is an early event evident in most radiation exposed patients observed within 2–4 months of treatment and leading to fibrosis later. Several cytokines and inflammatory molecules interplay in the vicinity of the tissue developing radiation injury leading to pneumonitis and fibrosis. While certain cytokines may be exploited as biomarkers, they also appear to be a potent target of intervention at transcriptional level. Initiation and progression of pneumonitis and fibrosis thus are dynamic processes arising after few months to year after irradiation of the lung tissue. Currently, available treatment strategies are challenged by the major dose limiting complications that curtails success of the treatment as well as well being of the patient’s future life. Several approaches have been in practice while many other are still being explored to overcome such complications. The current review gives a brief account of the immunological aspects, existing management practices, and suggests possible futuristic approaches.

## Introduction

Majority of cancer treatment involves the use of radiation, at least at times. Radiotherapy procedures involving lungs, as in the case of non-small cell lung cancer (NSCLC) and some others, face a major limitation in delivering higher radiation doses because of the concerns of later lung toxicities experienced in the form of pneumonitis and fibrosis. At early stages, the local inflammation and alterations in cytokine production, lead to pneumonitis development. In the following sections, the biomarkers of radiation-induced pneumonitis and current modalities under practice to overcome complications like radiation-induced pneumonitis available in literature will be discussed. Further, besides the recent progresses in developing modalities to reduce or mitigate radiation pneumonitis, the possibility of using HDAC inhibitors for the same will be highlighted.

## Immunology of Radiation-Mediated Lung Injury

Lymphocytes which perform diverse function in rendering immunity include T cells, B cells, and natural killer (NK) cells. T cells are known to act in antigen recognition and its processing while B cells are responsible for the production of cytokines and antibodies. NK cells can induce direct cell-mediated killing of virus-infected cells and tumor cells. This section focuses on the role of T lymphocytes and certain cytokines in the vicinity of tissue injured due to radiation exposure. In case of pulmonary tissues, γδ T cells reside in the subepithelium of alveolar and non-alveolar regions (1). As mentioned earlier, they play a key role in modulation of immune response against pathogens and allergens (2, 3). This is accepted now that lymphocyte subsets have distinct radiosensitivities, with immunosuppressive T regulatory cells being more radioresistant (4). Macrophages are relatively radioresistant and they are found in increased concentration in the vicinity of tumor stroma. This owes to their survival associated with recruitment (5–7). Simonian et al. explained the mechanism by which γδ T cells suppresses CD4^+^ cell recruitment by secreting regulatory IL-22 and help preventing progression of fibrosis (8). Different subsets of T cells perform specific functions. Certain studies have shown that T helper-9 subset may have a direct role in the asthma (9). In addition to the Th9, the Th22 and TFH cells have been investigated for their possible role in host defense against viruses and bacteria in the lung (10, 11).

In general, fractionated radiation therapy is considered immunosuppressive. Ceramide pathway activation occurs as a result of more than 10 Gy per fraction radiation in a sequential manner (12). Pulmonary fibrosis in lung cancer patients presents a picture where recovery of injury at specific tissue can be understood. It is observed that about 15% of patients receiving high-dose radiation for therapy of lung cancer exhibit pneumonitis (13).

Experiments performed under controlled environment *in vitro* have reported that cytokines such as transforming growth factor β (TGF-β) and interleukin-4 (IL-4) stimulate collagen synthesis in fibroblasts (14). While there are published evidences to establish the fact that certain cytokines like TGF-β1 play a role in progression of radiation-mediated fibrosis (15), IL-4, and IL-13 type 2 helper T cell (Th2) cytokines, in association with TGF-β are also known to facilitate fibrosis (16).

Investigators have proved that (17–19) elevation of TGF-β late during radiotherapy is associated with risk of pulmonary toxicity. To gauge the changes in levels of IL-1α and IL-6, a study was conducted by Chen et al. (20). It was noticeable that except TNF-α, there was a consistently elevated level of IL-1α and IL-6 prior to and throughout treatment in patients having radiation pneumonitis. However, levels of E selectin, L selectin, TGF-β1, and basic fibroblast growth factor (bFGF) did show some variation but were not correlated with radiation pneumonitis. Investigators correlated their study with Rübe et al. who observed different results as indication of correlation between radiation-induced pneumonitis in patients with NSCLC and serum levels of IL-6 or TGF-β prior to and after radiotherapy (21). TNF-α, is known to have its role in fibrosis development (22) and leads to TGF-β1 induction. Hence, it becomes a target molecule to check the progression of fibrosis.

Büttner et al. (14) in a similar study aimed to document the presence of IL-4 during the development of post-irradiation lung fibrosis. Male Fischer rats were irradiated with a single dose of 20 Gy and IL-4 expression in the irradiated lungs were monitored for a period of 3 months. IL-4 gene transcription as well as synthesis was increased in the irradiated lungs reaching a plateau concentration within 3 weeks after irradiation. Further, they showed a substantial IL-4 production by macrophages during development of post-irradiation lung fibrosis. These results suggest a correlation between local IL-4 protein expression and the development of radiation-induced pulmonary fibrosis (RIPF). With this kind of results, it was further noticed that IL-4 mRNA levels and the IL-4 protein levels do not closely correlate in the late stages of the development of pulmonary fibrosis (14). The results were in line with the other studies and could infer that it was an intracellular storage of IL-4 protein similar to the reported documentation of TNF-α in mast cells (23, 24). Regarding IL-1β, it is directly upregulated by radiation and activates other inflammation-related molecules such as the matrix metalloproteinases (MMPs), enzymes that regulate or degrade extracellular matrix components (25).

A similar study (26) was conducted to observe the changes of IL-6 during radiation pneumonitis, along with combined covariations of IL-6 and IL-10. However, in case of lung cancer radiotherapy, Crohns et al. found that after 3 months higher than baseline levels of IL-8 in serum and bronchoalveolar lavage (BAL) were associated with shorter survival (27). They could not establish any association between survival and the levels of TNF-α, IL-1β, IL-6, IL-12, and IL-18. A study conducted by Wilson et al. demonstrated that the severity of lung injury in mice was significantly decreased after mice IL-17A gene knockout, which proves the potent role of IL-17A in inflammation and fibrosis (28).

Haiping et al. moved a step ahead when he conducted an experiment to investigate whether radiation-induced pneumonitis in the mouse-irradiated lung could be prevented by recombinant adenovirus-mediated soluble TGF-β type II receptor gene therapy. They basically used an adenoviral vector expressing a soluble TGF-β type II receptor (AdCMVsTbR), which could bind to TGF-β and then block the TGF-β receptor-mediated signal transduction. After 4 weeks of irradiation, mice were killed and the concentration of TGF-β1 in the serum and BALF were measured. The researchers found that following thoracic irradiation with a single dose of 9 Gy, radiation-induced TGF-β1 release in the serum reached the first peak concentration at 12 h and then declined. They concluded that TGF-β plays a critical role in the pathogenesis of radiation-induced pneumonitis and that the interaction of TGF-β with its receptor is a promising prophylactic target (29).

Brickey et al. (30) carried out an investigation on the role of innate immune regulators like toll-like receptors in injury sustained from irradiation. In their study, they emphasized on the role of MyD88 in regulating innate immunity and nuclear factor kappa-B (NF-κB)-activated responses. The group examined the immune cells and factors during acute pneumonitic and fibrotic phases in MyD88-deficient animals receiving thoracic gamma (γ)-irradiation. Brickey et al. found that MyD88 supports survival from radiation-induced injury through the regulation of inflammatory factors that aid in recovery from irradiation (30, 31). Also, the absence of MyD88 resulted in unresolved pulmonary infiltrate and enhanced collagen deposition plus elevated Th2 cytokines in long-term survivors of irradiation.

These results summarily demonstrated that post-irradiation in lungs, MyD88 is important for regulating non-infectious inflammatory processes to promote tissue regeneration. Most notably, this group observed initially persisting cellular infiltrate and collagen deposition in the MyD88^−/−^ survivors of radiation lung injury in the context of diminished abundance of proinflammatory mRNA (i.e., Il6, Ccl2/Mcp1). This observation was correlated with results of other similar investigations (32, 33) who observed exacerbated lung injury and inflammatory cell accumulation in MyD88^−/−^ mice due to infection with pulmonary specific pathogens.

More recently, Wang et al. investigated the expressions of IL-17A in different phases of radiation-induced lung injury and the effect of dexamethasone. In their study, they observed that IL-17A expression was appreciable at 1 week, peaked at 4 weeks, and subsequently declined at 8 weeks after irradiation. However, on administering the drug of their choice, i.e., dexamethasone, IL-17A was reduced after application at all the observation periods. It was evident from this study that IL-17A plays an important role in the process of radiation-induced lung injury (34). The researchers further aligned their observation by comparing their study with other investigators who proved that IL-17A was significantly increased in a variety of chronic inflammatory disease models (35, 36).

**Figure 1:**
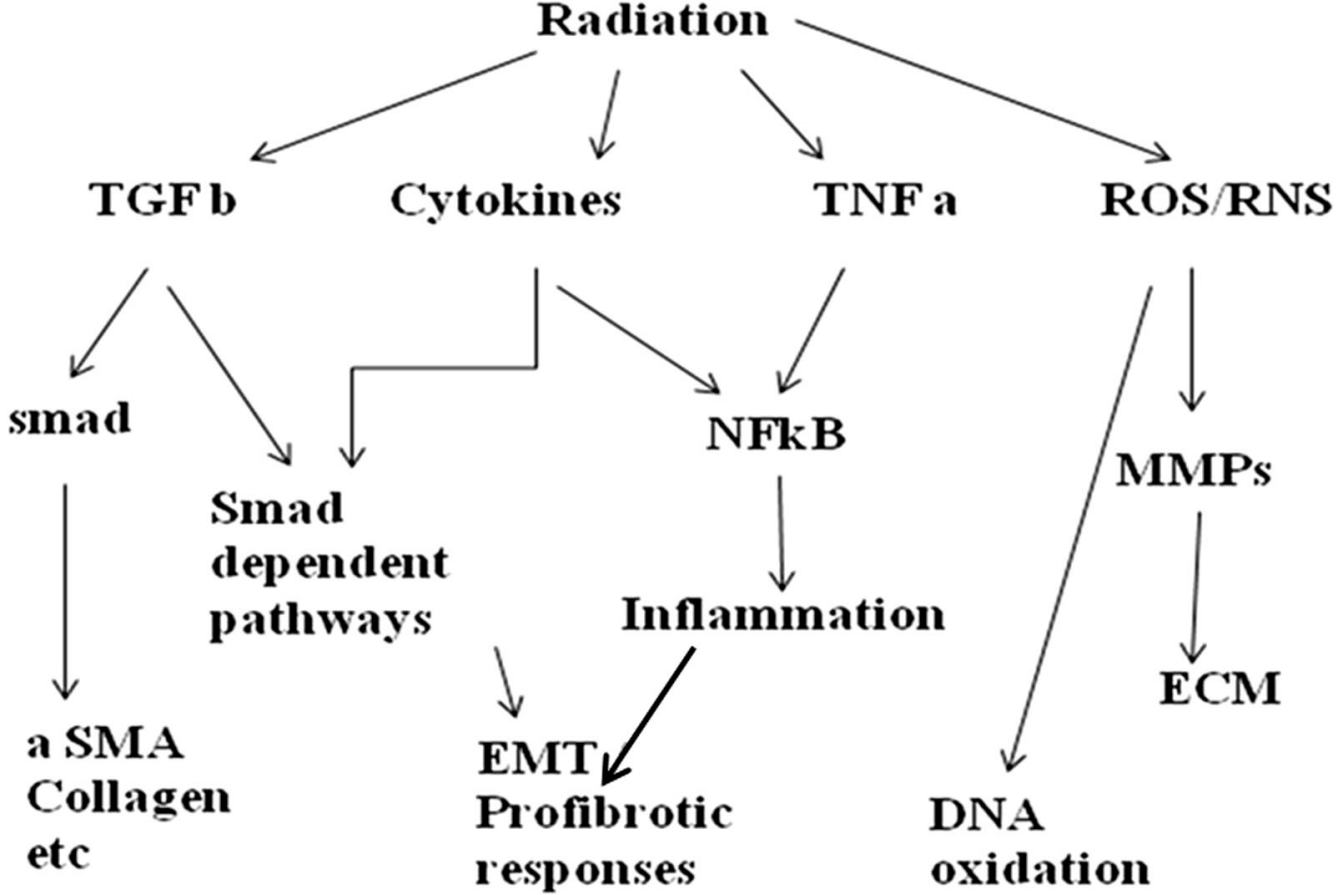
**Schematic diagram showing major signaling pathways involved in radiation pneumonitis**.

Finally, a generalized study needs to be mentioned here which aimed at exploring lung inflammation leading to pulmonary toxicity after radiotherapy in patients with NSCLC. This study by Shankar et al. showed that inflammatory cytokines were induced in NSCLC patients during and after radiotherapy. They recorded changes in levels of IP-10, MCP-1, eotaxin, IL-6, and TIMP-1 and could correlate it with higher-grade toxicity. Investigators reported that inflammatory cytokines were induced in patients with NSCLC both during and after radiotherapy and concentrations of IL-6, MCP-1, MCP-3, and IP-10 correlated with the mean lung dose (MLD) (37). This particular study and so far quoted studies suggest the induction of different inflammatory cytokines as a result of radiation exposure which develops into radiation pneumonitis. These particular cytokines play a specific role in initiation and development of fibrosis and pneumonitis. Not only can they be targeted as an intervention point, but also may be exploited as biomarker for early detection of radiation-induced lung injury. Figure 1 is a schematic presentation of some important signaling pathways playing role in radiation-induced pneumonitis.

## Predictors for Radiation-Induced Lung Toxicity

Radiation-induced pneumonitis is characterized mainly by increased infiltration of neutrophils and macrophages, enhanced activity of E selectin, tumor necrosis factor (TNF), and monocyte chemotactic factors. The inflammatory condition in the local region results into acute inflammation leads to radiation-induced fibrosis, if untreated. Understanding of molecular and cellular processes helps in defining biomarkers that identifies the risk for developing radiation pneumonitis. For preventive measures and to reduce the severity of radiation-mediated injury, it becomes import to identify and define certain biomarkers and predictors of radiation-induced pneumonitis.

### Matrix Metalloproteinases (MMPs)

Matrix metalloproteinases, a family of proteolytic enzymes involved in the turnover and remodeling of basement membrane and extracellular matrix proteins, can be considered as potential predictor of lung tissue injury (38). Gelatinase B and type IV collagenase degrades macromolecules like fibronectin, collagen (type IV), laminin, and elastase and in the lung interstitial matrix. Tissue inhibitors of metalloproteinases are generally required to regulate the activity of MMP and form physiologically irreversible complexes. One of the MMPs, MMP-9, is produced by different cell types like antigen-presenting cells, macrophages, and blood cells-like eosinophils and neutrophils, and holds the capacity to digest basement membrane. It would be too early to reach to the conclusion that MMPs realistically and evidently correlate with the occurrence of radiation-induced pneumonitis. Measurement of MMP-3 and MMP-9 were observed not to correlate with radiation fibrosis (39). Although the levels of certain MMPs increase during radiation lung injury (40) the role of these molecules still remains unclear.

### Transforming Growth Factor

Transforming growth factor α is a protein coded by the TGFA gene in humans and is a member of epidermal growth factor having mitogenic properties. TGF-α is known to induce phenotypic modulation of human lung fibroblasts to myofibroblasts (41, 42) and is a potent stimulator of collagen synthesis (43). It is reported that TGF-α gene expression increased at 1–14 days after radiation therapy simultaneous to the changes in fibroblast gene expression of collagens I/III/IV and fibronectin (44, 45). As discussed earlier that TGF-α is produced by different cell types, pneumocytes, and also fibroblasts. The constant abnormal level by the end of the treatment indicates the level of injury in the local tissue. As the biomarker for radiation pneumonitis, plasma TGF-α1 levels have been successfully used for the stratification of patients into low-, intermediate-, and high-risk groups (44, 45). However, lung cancer patients often have elevated plasma TGF-α1 level because of tumor TGF-α1 production (46). A decrease in TGF-α1 expression may therefore occur as the tumor regresses. Serial plasma TGF-α1 determination and dose–volume relationships are needed to identify patients at low risk (47). TGF-α1 is secreted as a biologically inactive complex that is activated by various elements such as matrix proteins and proteases in response to tissue damage (48, 49). In an experimental setup, where radiation-sensitive and non-sensitive strains were produced, it was observed that TGF-α and latent TGF-β1 activation occurred by DNA-damaging agents and free radicals produced due to radiation therapy (50). This activation of TGF-α and latent TGF-β1 in plasma is suggestive of initiation of radiation pneumonitis after radiation therapy. Notable reduction in the fibrotic proliferation was observed in rats treated with an adenoviral-mediated soluble TGF-α type II receptor (51). The predictive value for the initiation and progression of interstitial pneumonitis was 90% or more when pre-transplantation plasma TGF-β1 levels were more than two SDs above the mean established in the controls. In an investigation, absolute TGF-β plasma levels did not differ between the groups of patients without or with pneumonitis. The patients who developed pneumonitis clearly showed increase in TGF-β levels in the middle of the radiation therapy course relative to their pretreatment levels while TGF-β plasma levels of patients who did not develop pneumonitis decreased over the radiation treatment. The difference in the relative TGF-β dynamics between the groups reached marginal significance in the third week of the treatment but weakened toward the end of the radiation therapy course. Based on the test’s ability to yield more accurate estimate of complication probability in an individual patient compared to empirically expected probability in similar group of patients, the utility of TGF-β testing was evaluated at each radiation therapy week. The accuracy of prediction deteriorated at later time points (weeks 4–6) rendering the end-radiation therapy ratios without predictive power (52). The predictive value of TGF-β1 on lung toxicity in patients with advanced breast cancer treated by high-dose chemotherapy and autologous bone marrow transplantation has been assessed and documented earlier (53). Hence, TGF-β is regarded useful not only as a marker for pneumonitis and fibrosis but also predicting an individual patient’s risk for developing late radiation-induced normal tissue injury. To establish a direct correlation of TGF-β1 with the occurrence of pneumonitis, an observation was made on patients. Then, 80% of patients who developed signs and/or symptoms of pulmonary injury consistent with pneumonitis had persistently elevated plasma TGF-β levels by the end of therapy. On the contrast, three patients who did not showed sign and/or symptoms of pneumonitis, plasma TGF-β levels normalized by the end of radiotherapy. This finding appeared to be independent of the volume of irradiated lung. The investigators thus concluded that their preliminary results suggested that the failure of plasma TGF levels to return to normal, before the completion of radiotherapy with curative intent for lung cancer, might be predictive for an increased risk of pneumonitis development. However, apart of the pneumonitis, it was hypothesized that TGF-β appears to be produced either directly or indirectly by some NSCLC, and declining plasma levels during therapy may be indicative of tumor cell death (17). Early circulatory chemokines thus provide a good predictive measure to identify the individual at risk of developing radiation pneumonitis.

### IL-6

IL-1 and IL-6 are cytokines responsible for mediating inflammatory responses (54). TNF-α is known to promote the growth of fibroblasts and stimulate the synthesis and release of cytokines such as IL-6 and IL-1. The cascade effect of cytokines follows after that (55). IL-6 synthesized and secreted by various cells in the lung parenchyma is a pleiotropic cytokine that regulates immune responses and inflammatory response by inducing hepatocytes and lung fibroblasts to release acute phase proteins (56, 57). Hence, understandably, IL-6 levels in the serum can be used to judge the inflammatory state of the lung. Serial plasma specimen analysis for circulating cytokine changes prior to, during and up to 12 weeks after radiation in 24 patients diagnosed with radiation pneumonitis was done to ascertain cytokine levels after thoracic radiotherapy. Assessment of IL-1 α, IL-6, MCP-1, E selectin, L selectin, TGF-β1, and bFGF during the progression of disease revealed some promising information. However, no significant correlation between variation in pattern of changes of MCP-1, E selectin, L selectin, TGF-β1, and bFGF and radiation pneumonitis could be established. The study summarized as high pretreatment plasma levels of IL-6 predisposed patients to the risk of radiation pneumonitis and therefore pretreatment IL-6 level may serve as a predictor for radiation pneumonitis. Patients with radiation pneumonitis demonstrated higher circulating levels of IL-6 during and immediately after thoracic radiotherapy. However, the mechanism for IL-6 involvement in radiation pneumonitis warrants further investigation (58). Variation in IL-6 was also observed by a Chinese group (59), where it increased after RT. However, in the same study, a decrease in IL-10 was also observed, which indicates that RT induces changes in IL-6 and IL-10.

### Platelet-Derived Growth Factor (PDGF)

Platelet-derived growth factor has importance in showing profibrotic activities. Many fibrogenic mediators like IL-1, TNF-α, bFGF, and thrombin are known to accelerate and take part in the fibrotic process. PDGF further has role in proliferation and migration of myofibroblasts during fibrosis. In a study, it was observed that irradiation of the endothelial cells or human cancer cell lines like A549 cells induced phosphorylation of PDGFR in fibroblasts at 6 and 72 h after irradiation resulting in activation of PDGFR and subsequent infiltration of myofibroblasts in the local area. Thus activated fibrogenic mediators become an important biomarker for an individual at risk of radiation-induced pneumonitis during 6 and 72 h after irradiation. Also, all PDGF isoforms were significantly upregulated after 10 Gy radiation in human lung microvascular endothelial cells (P<M0.02) and persisted up to 72 h after irradiation (60). Fibrotic activity of TGF-α and bFGF depends on PDGF profibrotic activity. Radiation-induced expression of PDGF and phosphorylation of PDGF receptors persist at least till 72 h after irradiation and till late phase in radiation-induced fibrosis.

**Figure 2:**
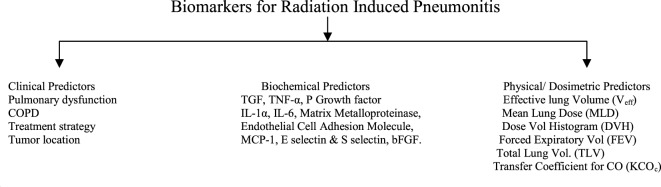
**Schematic diagram depicting broad categories of predictors for radiation-induced lung toxicity like pneumonitis and fibrosis**.

The occurrence and severance of radiation lung toxicity is also dependent upon different factors such as age, smoking habits, location of tumor, and lung dosimetric factors. Out of these general factors, the lung dosimetric factors and the lung dose–volume histogram (DVH)-based normal tissue complication probability (NTCP) models have been extensively studied. Point parameters such as volumes (*V*) receiving a *V*30, *V*20, *V*13, *V*5, or other dose, the dose to a certain portion of the lung volume (such as *D*30), effective lung volume, and MLDs are of immense importance. Investigation group of Kong and coworkers have provided an extensive review on these parameters (61–63). Though none have optimal predictive power (for individuals) for routine clinical use, various NTCP models and many DVH parameters are still considered as predictive of the risk of radiation pneumonitis for populations of patients. Rather the investigators found the predictive capacity to be individually varying. Precisely, they quoted that statistically significant association or description of complication rates for populations of patients is not equivalent to a good predictor of toxicity for each individual patient. They further elaborated the conclusion with example. *V*13, *V*20, and MLD were all predictive of radiation pneumonitis, but they all had a similar suboptimal predictive ability for grade 2 and higher RIL (64). If cut-offs are 30% for *V*20, 20 Gy for MLD, and 10% for NTCP, these factors have positive-predictive values of only 50–71% and negative-predictive values of 85–89% (61, 62, 64). The cut off values used in this study generated reasonable (80–85%) certainty of identifying low-risk patients but unacceptable (50–70%) low-sensitivity and positive-predictive values. Figure 2 depicts predictors of radiation-induced pneumonitis and fibrosis.

### Dosimetric Parameters [Pretreatment Performance Status and Forced Expiratory Volume (FEV)]

The *V*–dose parameter is defined as the percentage of computed tomography (CT)-defined total lung volume minus the PTV receiving a higher or equal dose compared to the threshold dose (e.g., 20 or 25 Gy). The MLD is defined as the average dose of the CT-defined total lung volume.

A set of samples were reviewed in a study to identify factors that may predict for severe radiation pneumonitis or pneumonopathy (RP). In 144 evaluable patients, 12 (8.3%) experienced severe RP. The most significant factor predicting for severe RP was performance status (*p* < 0.003). The risk of severe RP was 16% for PS-1 patients vs. 2% for PS-0 patients. On the basis of gender, women were found to be significantly more susceptible to develop severe RP than men (*p* < 0.01). FEV of the lung in 1 s (FEV1) was also significant (*p* < 0.03). To find the possibility of radiation dose to be used as biomarker for radiation pneumonitis, median radiation dose and median initial radiation were assessed. Median radiation dose was 59.2 Gy and median initial radiation field size was 228 cm^2^. Neither radiotherapy factor predicted for RP. The study also investigated chemotherapy drugs and schedule which also were not found significant predictors for severe radiation pneumonitis. Evidently, it was concluded that pretreatment performance status, gender, and FEV1 are significant predictors of severe radiation pneumonitis (65).

There are further comprehensive and reliable studies conducted to establish effective volume (*V*_eff_), total lung volume mean dose, and location of tumor as potential predictors for radiation pneumonitis. With an aim of identifying a clinically relevant and available parameter upon which to identify NSCLC patients at risk for pneumonitis, univariate analysis was done. The percent of the total lung volume exceeding 20 Gy (*V*20), the *V*_eff_ and the total lung volume mean dose, and location of the tumor primary (upper versus lower lobes) to be statistically significant relative to the development of >grade 2 pneumonitis. Multivariate analysis was done in the same study which further revealed the *V*20 to be the single-independent predictor of pneumonitis (66). Complete vetting of the study presented the fact that the *V*20 from the total lung DVH is a useful parameter easily obtained from most 3D treatment planning systems. Authors could concluded that the *V*20 may be useful in comparing competing treatment plans to evaluate the risk of pneumonitis and may also be a useful parameter upon which to stratify patients or prospective dose escalation trials. There is strong evidence that both the *V*20 and the MLD, correlate with the risk of high-grade radiation pneumonitis (66, 67). It was concluded by a group of researchers that both the biological and physical risk factors allow better identification of patients at risk for the development of symptomatic radiation-induced lung injury (68).

Several other factors are being investigated for being early biomarkers for pneumonitis. Age, WHO performance status, and tumor locations are potential risk factor for which conflicting evidence has been published and discussed. As mentioned earlier, the range of factors spans from clinical to biochemical/molecular to physical. In one such study, several clinical factors were investigated. Pulmonary dysfunction before radiation therapy may predispose patients for radiation pneumonitis. Chronic obstructive pulmonary disease (COPD) and impaired lung function measurements have been documented to be associated with radiation-induced lung toxicity (65, 69). While in other studies, it was found to have no statistically significant relationship (70, 71). Thus there are diverse studies with varying results and with no significant audacity it can be said that these are predictors for radiation-induced lung toxicity. Also, it doesn’t apply for every individual.

### Dosimetric Predictors

The records of 160 patients who received radiation therapy for NSCLC were reviewed by Jenkins and Watts to investigate whether multiparametric models that incorporate clinical and physiologic factors have improved accuracy (72). Patients treated with the same dose and with an identical technique were analyzed for dosimetric, pulmonary function, and clinical parameters to determine their ability to predict for the subsequent development of radiation pneumonitis. On univariate analysis, fractional volume of lung receiving >5–20 Gy, absolute volume of lung spared from receiving >5–15 Gy, MLD, craniocaudal position of the isocenter, transfer coefficient for carbon monoxide (KCOc), total lung capacity, coadministration of angiotensin-converting enzyme inhibitors (ACEIs), and coadministration of angiotensin-receptor antagonists significantly correlated with the risk of pneumonitis. The authors were able to define a new parameter termed transfer factor spared from receiving >5 Gy (TFS5) by combining the absolute volume of lung spared from receiving >5 Gy with the KCOc. TFS5 represents a simple parameter that can be used in routine clinical practice to more accurately segregate patients into high- and low-risk groups for developing RP.

Several reports have associated dose–volume metrics with radiation pneumonitis (66, 73). Hence, it is clear that radiation pneumonitis is correlated with the dose delivered to a particular fractional volume of the lung.

### Single Nucleotide Polymorphism (SNP)

Single nucleotide polymorphism is a change in which a single base in the DNA differs from the usual base at that position. Researchers conducted a study in which they combined a genome-wide SNP-associated evaluation of inbred strain response with prior linkage and gene expression data to identify genes which influence the fibrotic response to thoracic cavity radiotherapy. It was inferred from the study that on combining genomic approaches, variation were identified within specific genes which function in the tissue response to injury as associated with fibrosis following thoracic irradiation in mice (74).

### Other Markers

Krebs von den Lungen-6 (KL-6) is a mucinous high molecular weight glycoprotein, expressed on type 2 pneumocytes. It is generally regarded as the lung epithelium-specific protein KL-6. Previous studies have suggested that KL-6 is a useful marker for the clinical diagnosis of pneumonitis (75). KL-6 is chemotactic for human fibroblasts and has functional role in fibrotic process. There are published reports suggesting serum KL-6 level reflects the severity of radiation pneumonitis, and the increase (>1.5-fold) was associated with serious radiation pneumonitis that was refractory to steroid therapy (76).

Very few studies were comprehensive enough and to be mentioned where BAL has been tested as a biomarker for clinical pneumonitis.

### Reactive Airway Disease

Reactive airway disease has been identified as a predictive factor for pulmonary toxicity in the patients who were administered the radiation therapy for the treatment of NSCLC (77).

### Age

Studies have shown that patients of 65 years of age or less are more prone to the toxicity than the old age fellows. It is inferred in the study that the reason for this factor is the administration of split course therapy (77).

### Normal Tissue Complication Probability (NTCP)

Computed tomography-based NTCP has been resulted to be the best predictor for the development of the pulmonary symptoms and the NTCP values in patients with pre-RT PFTs have been found to be remarkable (78).

### CYFRA 21-1

Cytokeratin 19 fragment has been considered as marker of apoptosis in including lung cancer and other cancer types. There may be chances that cytokeratin 19 might be released by type II pneumocytes during radiation-induced epithelial cell damage as it is believed to play an important role in apoptosis of type II pneumocytes during radiation-induced lung tissue damage (4).

### Intercellular Adhesion Molecule-1 (ICAM-1)

Intercellular adhesion molecule-1 belongs to the immunoglobulin group and can be induced by interleukin 1 and TNF. The recruitment and activation of lymphocytes occur in the pathogenesis of radiation-induced pneumonitis. ICAM-1 assists in the leukocyte accumulation due to its adhesive characteristic. The vascular endothelial cells, epithelial cells, and lymphocytes are found etched with the ICAM-1 protein. Studies suggest that on comparing the serum levels of ICAM-1 in healthy individuals to those of cancer patients, it was higher in latter, irrespective of the development of pneumonitis. It has been considered in the findings that radiation-induced pneumonitis may be detected in its early phases with the use of serum ICAM-1 marker, however, monitoring has to be scheduled prior to and throughout the therapy (4).

### Central Lung Distance (CLD)

Central lung distance has been used as a parameter to calculate the percentage of lung irradiated for tangential field. A study for the carcinoma of the breast depicts that the higher CLD amounted to higher irradiation and it suggests CLD as the best method of calculating the irradiation of lungs (79).

### Serum Amyloid

Serum samples were analyzed in a study from the serum of stages Ib–IV lung cancer patients who developed radiation pneumonitis after a year of radiation therapy. Serum amyloid A was found to be useful auxiliary marker in this finding for predicting the severity of radiation pneumonitis (80, 81).

## Current Treatment Strategies

Tissue toxicity in lungs and its progression in local/regional areas require specific treatment that is also decided by the physiological status of subject. The most common form of radiation pneumonitis is referred to as bronchiolitis obliterans organizing pneumonia (BOOP) is an inflammatory response of the tissues. Pneumonitis is sub-categorized into organizing pneumonia and secondary organizing pneumonia and the term BOOP is used for the non-idiopathic forms such as radiation therapy BOOP (82) which is characterized by a specific pulmonary lesion with a typical pathologic pattern. The treatment (chemotherapy) for radiation pneumonitis, BOOP, and other radiation-induced lung tissue damage include steroids and diuretics, corticosteroids, other hormones, enzymes, redox regulators, and antioxidants. These molecules have been tested solely or in combination to obtain better prognosis. Criteria which are critical for obtaining recovery and understanding the mode of action of particular drug includes type of drug, doses of drug, period of treatment, time of initiation of drug, and the condition of patient and the irradiation type (whole chest irradiation/chest wall irradiation/partial irradiation or hypofractionated irradiation).

### Steroids and Corticosteroids

Prednisone, a synthetic corticosteroid drug with immunosuppressant properties, has widely been used in different inflammatory diseases like radiation pneumonitis and some autoimmune diseases but is known to have adverse effects. Prednisone gets converted *via* hepatic metabolism to prednisolone which improves lung function and minimizes symptoms in radiation-induced pneumonitis and lung tissue toxicity (83). An isoflavonoid, genistein derived from soy products, is also known to inhibit tumor growth by enhancing apoptosis. In mice at non-toxic doses, it protects against radiation-induced lung damage (84).

### Pentoxifylline (Trental)

Pentoxifylline, a methylxanthine derivative, inhibits phosphodiesterase and improves blood flow by increasing erythrocyte and leukocyte flexibility and inhibiting platelet aggregation. Pentoxifylline also stimulates cytokine production. It has shown encouraging results in preventing early and late lung toxicity (85). The current National Comprehensive Cancer Network guidelines recommend concurrent full-dose cisplatin-based chemoradiotherapy regimens. Weekly, docetaxel with conventional radiotherapy resulted in 47% grade 3 or higher-grade pneumonitis, including 19% fatal pulmonary toxicity. Pentoxifylline acts as a competitive non-selective phosphodiesterase inhibitor, raises intracellular cyclic adenosine monophosphate (cAMP), activates cAMP-dependent protein kinase, inhibits TNF, and leukotriene synthesis. In the typical conditions like radiation pneumonitis, it has been used as suppressant of innate immunity. In treating breast cancer, chemotherapy concurrent with radiation also plays significant role in developing radiation pneumonitis. Concurrent medication with tamoxifen is among the several factors that culminate into radiation pneumonitis after radiation therapy for breast cancer (86).

### Angiotensin-Converting Enzyme Inhibitors

By causing vasoconstriction, angiotensin increases blood pressure and hence is frequently being used to treat such cases. Angiotensin is an oligopeptide hormone derived from the precursor molecule angiotensinogen, a serum globulin produced in the liver. ACEI causes relaxation of blood vessels and decrease blood volume leading to lower blood pressure. The drug thus is a potent treatment option in conditions like hypertension and elevated blood pressure. ACEIs molecules such as ramipril, perindopril, captopril, and enalapril have been investigated in trial for long.

A study by Wang and coworkers in Texas on whether ACEIs reduce the risk of symptomatic radiation pneumonitis in patients with NSCLC after definitive radiation therapy showed the trend toward reduction in symptomatic radiation pneumonitis among patients taking ACEIs during radiotherapy for NSCLC was not statistically significant. However, this was observed that certain subgroups may benefit from use (i.e., male patients and those receiving low MLD) (80, 81).

### Adjuvant Systemic Chemotherapy

Post-operative local/regional radiotherapy (PORT) is generally considered as curative for patients suffering from lung cancer but may cause subsequent inflammatory responses and damage the tissue. Keller et al. (87) conducted a randomized trial of postoperative adjuvant therapy in patients with completely resected stage II or IIIA NSCLC. It was encouraging to know through that adjuvant systemic chemotherapy alone was effective in treatment compared to treatment in combination with radio therapy. Further, concurrent cisplatin-based chemotherapy in addition to PORT did not improve local-regional control or survival compared with PORT alone (87). However, later (88, 89), it was reported that effectiveness of adjuvant systemic chemotherapy using different set of molecules like paclitaxel and carboplatin. A 50.4 Gy irradiation in 28 fractions in 6 weeks was done by Bradley et al. (88) followed by a boost of 10.8 Gy in six fractions for extracapsular nodal extension. Decrease in percentage of survival rates for 1, 2, and 3 years (86, 70, and 61%, respectively) was observed with no notable difference in survival between stages II and IIIA NSCLC patients. However, the 1-, 2-, and 3-year survival percentage were promising. Feigenberg obtained almost similar results showing survival of 72 and 44% for 2 and 5 years. The results obtained indicated the synergistic role of adjuvant therapy along with radiotherapy. However, more such studies are warranted to establish adjuvant therapy as a reliable treatment approach. The current National Comprehensive Cancer Network guidelines recommend concurrent full-dose cisplatin-based chemoradiotherapy regimens (90). Onishi et al. (91) investigated on concurrent two-dimensional radiotherapy and weekly docetaxel in the treatment of stage III NSCLC. Although there was a notable local response using docetaxel, the survival rate was not significant enough. Weekly, docetaxel with conventional radiotherapy resulted in 47% grade 3 or higher-grade pneumonitis and 19% fatal pulmonary toxicity (91).

### Indigenous and Traditional Formulations

It was documented in a study that in comparison to the control group treated only by antibiotic and hormone pulse therapy, Shenqi fuzheng injection (a traditional Chinese medicine) in combination with antibiotics and short-term pulse therapy with hormones had significant effect in improving radiation pneumonitis. The combination improved the radiation-induced lung injury probably by controlling regulation of subsets of T lymphocytes like CD4^+^, CD8^+^, and CD4^+^/CD8^+^ ratio (92). Variable results and limited studies on indigenous drugs, however, have made clinicians reluctant to implicate them as mainstay treatment approach. An indigenous Chinese herbal formulation composed of Liangxue Jiedu Huoxue Decoction formulation comprised of seven herbs namely, *Radix astragali, Radix rehmanniae, Cortex moutan, Peach seed, Rhizoma chuanxiong, Fructus forsythia*, and *Flos carthami*, was tested against 100 lung cancer patients scheduled to receive radiotherapy. Not only the lung injuries were limited and improved in treatment group, encouraging results were obtained showing lowered incidence rate of radiation pneumonitis in the treatment group than in the control group receiving only radiotherapy (13.04 and 33.33%, respectively).

### Oxygen Therapy

Although this is not a curative therapy, it is more like a support. The subject undergoing the treatment for lungs-related complications often suffers from breathlessness. The oxygen therapy hence provides an associated support in combination with drugs. It helps in bringing homeostasis of the individuals by leveling the oxygen in the blood and in tissues. However, it has no direct and definite curative properties in terms of inflammation.

### Molecular Approaches

Apart of the chemotherapy, studies have been conducted to ascertain the role of selected genes in modulating the pathologic conditions like RIPF. It was observed that a combined deficiency in both Tlr2 and Tlr4, and not alone, leads to enhanced radiation-induced fibrosis in the C57BL/6 mouse model irradiated with 18 Gy single dose in the thoracic region (93). Research shows the prominent target areas are either the intermediates of the inflammatory pathways or the proteins/enzymes catalyzing biological reactions in-between in the pathogenesis of radiation-induced pneumonitis and fibrosis. Whole process of inflammation leading to radiation-mediated pneumonitis to the severe and chronic form of radiation-induced fibrosis has several potent targets for treatment. The process starts from induction of TGF-β by radiation exposure and ends up with unbound snail migrating to the nucleus and repressing E cadherin leading to a mesenchymal-like phenotypic change. In between of these two major events, there are molecules like NRF2, Smad binding element, E cadherin, and GSK3 β which can be studied as intervention points. Inhibitors of TGF-β RI like SM16 have capability to reduce the severity of radiation-induced lung injury. Protection against development of such injuries has been documented in a rat model (94).

The human *TGFβ1* gene is located on chromosome 19q13.1–13.39. Associations between the *TGFβ1* 869C/T polymorphism and susceptibility to radiation pneumonitis have been documented earlier (95, 96). A comprehensive meta-analysis in this regard reports that there was a significant association between *TGFβ1* 869C/T polymorphism and RP susceptibility (OR = 1.77; 95% CI, 1.27–2.47; *p* = 0.0007). However, the meta-analysis included fewer studies and small ethnic group (97).

A significant survival advantage could be obtained by subsiding p-Smad2 and p-Smad1 expression. Suppressed expression of p-Smad2 and p-Smad1 leads to lowered expression of genes responsible in canonical and non-canonical TGF-β signaling which results in reduced inflammation and pulmonary fibrosis (98). Investigators have pointed TGF-β/Smad as most effective target for development of different inhibitors (99, 100). Fluorofenidone [1-(3-fluorophenyl)-5-methyl-2-(1H)-pyridone, AKFPD], a novel pyridone antifibrotic agent, reduced cardiac and kidney fibrosis by inhibiting connective tissue growth factor expression (101, 102). Cytokines and growth factors thus are oozing as hotspots in checking inflammatory responses. Sivelest at, a neutrophil elastase inhibitor, decreases collagen deposition and aggregation of neutrophil in the tissues of lungs and improves the lung injury be directly interfering in the development of inflammatory response. A brief overview of the current mainstay treatment strategies and strategies under investigation are given in Tables 1 and 2.

**Table 1:** **A summary of current available approaches for radiation pneumonitis management**.

Steroids and corticosteroids	– Mainstay of treatment. – Administered in primary pneumonitis. – Oral administration. – General dose: prednisolone 1 mg/kg (max 60 mg) for a period of 2 weeks. – Followed by slow tapering over weeks. – Incomplete course leads to worsening of symptoms. – Acts as an anti-inflammatory substance.
Angiotensin-converting enzyme inhibitors	– Effective in mitigating radiation-induced pneumonitis. – Trails on rats showed variable success. – Limited efficacy in human subjects. Ex: enalapril. – Certain conditions like bilateral renal artery stenosis required to be ruled out before initiation of therapy. Blood pressure should be in control. – Acts by decreasing vascular remodeling and levels of transforming growth factor β (TGF-β).
A usual and in practice approach	Once the differentials are ruled out, start the patient on prednisolone 1 mg/kg. Continue prednisolone for 2 weeks followed by tapering over 2 weeks. Start pentoxifylline 400 mg thrice daily and enalapril 2.5 mg twice daily for 2–4 weeks. Consider stopping radiotherapy for a few days if highly symptomatic. If no improvement of symptoms, consider other immunosuppressant-like azathioprine (103).
Molecular approaches	Several intermediates that take part in the development of inflammatory response in the lungs can be targeted. Studies have been done on such targets, anti-TGF-β type 1 receptor (104), DNA intercalator, inhibits cell proliferation (105), immunoregulation, restoration of the immunological balance and inhibitor of neutrophil elastase (106), decreases collagen deposition and the accumulation of neutrophils. Those are not yet in clinical practice.
Adjuvant systematic chemotherapy	– Research shows effectiveness when administered alone. – Paclitaxel and carboplatin have shown encouraging results. – National Comprehensive Cancer Network guidelines recommend concurrent full dose. – Cisplatin-based chemoradiotherapy. – More studies needed to be done before establishing it as reliable therapy.

**Table 2:** **Investigations on application of different treatment regimens for radiation-induced lung complications**.

S. no.	Inflammatory response/pathologic condition	Reference	Treatment dose	Radiation dose	Period of course	Drug type
1	Pneumonitis	(107)	Adriamycin: 50 mg/m^2^ rechallenged for two further cycles with no symptoms with steroid cover	15 Gy	2 months	Steroid
2		(108)	Adriamycin: 30 mg/m^2^	36 Gy	3 weeks	Steroids
3		(108)	Adriamycin: 30 mg/m^2^	59.4 Gy	6 weeks	Steroids
4		(109)	Paclitaxel: 175 mg/m^2^ rechallenged for one further cycle with no recurrence after premedication with steroids	43.2 Gy	12 days	Not specified
5		(110)	Intrapleural instillation of 30 mg of adriamycin	36.5 Gy	1 month	Steroids and diuretics
6		(111)	Mn porphyrin, MnTE-2-PyP5^+^ (6 mg/kg/24 h)	28 Gy	2 weeks	Antioxidant and redox-modulating Mn porphyrin
7		(112)	Genistein diet (10 mg/kg) (in combination with radiation)	10 Gy	9 fractions of 3.1 Gy over 30 days	Soy isoflavone
8		(113)	Omeprazole, esomeprazole, and lansoprazole	Radiation regimen was reformulated after 50 Gy if the lesion was extended	7 days	Proton pump inhibitors
9		(114)	Pentoxifylline (400 mg orally)	40–84 Gy	8 weeks	Anticytokine
10	Stages I–III NSCLC after radiation therapy	(80, 81)	Angiotensin-converting enzyme inhibitors (ACEIs)	60 Gy	During the entire course of RT (not specified for individual patient)	ACEIs
11	Radiation pneumonitis/radiation-induced fibrosis	(115)	Cyclooxygenase-2 inhibitor twice daily	13.5–14.75 Gy	40 consecutive days from the day of local thoracic irradiation or 40 or 80 days later	Celecoxib (an inducible enzyme)

## Future Prospect

Radiation-induced pneumonitis results from prolonged inflammation caused in the lung tissue due to irradiation. Any approach capable of reducing the inflammatory response or increasing anti-inflammatory molecules thus can be useful in managing radiation-induced pneumonitis. In the recent past, our laboratory has been involved in the development of mitigators of radiation injury, mortality in particular, against whole body irradiation in mice model. As a hypothesis, it was processed that inhibitors of histone deacetylases may be used as mitigators of radiation injury (116, 117). We have shown survival advantage against lethal whole body irradiation using trichostatin A (TSA) and epigallocatechin gallate (118), and diallyl disulfide (DAS) (119). Sulforphane, another dietary HDAC inhibitor, was observed to reduce radiation-induced micronuclei in human peripheral blood lymphocytes (116, 117, 120). Sodium butyrate (NaB) has been shown to reduce inflammatory cytokine IL-1 (121) and renders neuroprotective effect in mice and similarly a number of HDAC inhibitors having anti-inflammatory properties have been described by Losson et al. (122). Recently, Hu et al. (123) have shown epigenetic regulation of IL-6 by changing HAT and HDAC dyanamics in a paraquat-induced pulmonary fibrosis model. Yet in another study (124) using peripheral blood mononuclear cells from type II diabetes mellitus (T2DM) patients, designing of future anti-inflammatory drug against T2DM using inhibitors targeted against HDAC 3 has been postulated. The ability of TSA in attenuating bleomycin-induced lung injury in mice has also been demonstrated (125) recently. The HDAC inhibitors used in our studies were found to significantly reduce radiation-induced inflammatory cytokines such as IL-6, TNF-α, TGF-β in mice plasma, and serum. For example, TSA effectively reduced IL-6 and TNF-α while enhanced IL-5 level where as DAS reduced IL-6 and TGF-β while increased G-CSF levels. Both the compounds have rendered significant enhancement in survival of lethally irradiated mice and possess no detectable toxicity and hence those compounds further may be explored for their beneficial application in radiotherapy in order to overcome radiation-induced lung injuries including pneumonitis and fibrosis. However, no experimental studies have been performed in this direction so far by our group or others in appropriate model systems for radiation pneumonitis or fibrosis.

## Author Contributions

AK, TH, MT, NG, and PA participated in compilation and editing of the manuscript. PA, SP, MT, and HP participated in literature and data collection. All authors read and approved the final version.

## Conflict of Interest Statement

The authors declare that the research was conducted in the absence of any commercial or financial relationships that could be construed as a potential conflict of interest.
